# Echoendoscopy with elastography in mediastinal lymph nodes

**DOI:** 10.31744/einstein_journal/2019MD5157

**Published:** 2019-11-27

**Authors:** Rogerio Colaiacovo, Altair da Silva Costa, Gustavo Andrade de Paulo, Silvia Mansur Reimão, Marco Antonio Ribeiro Camunha

**Affiliations:** 1 Hospital Israelita Albert Einstein, São Paulo, SP, Brazil; 2 Escola Paulista de Medicina, Universidade Federal de São Paulo, São Paulo, SP, Brazil

**Keywords:** Mediastinum, Biopsy, fine-needle, Endoscopic ultrasound-guided fine needle aspiration/methods, Ultrasonography, interventional, Lymph nodes, Bronchoscopy

## Abstract

Elastography is a widely used procedure in conventional ultrasonography that has recently been incorporated in echoendoscopy. This is an innovative and promising technology that aims to increase the negative predictive value of endoscopic ultrasonography and fine-needle aspiration punctures. It is useful for directing punctures in suspect areas and, consequently, improves diagnostic performance. This is a non-invasive technique, easy to perform, without additional costs or complications. The main indications are the analysis of solid pancreatic masses, lymph nodes, subepithelial lesions, lesions in the left hepatic lobe and in the left adrenal. Negative or inconclusive cases of fine-needle aspiration can be submitted to elastography when there is a strong suspicion of malignancy. Elastography has a high precision for the differential diagnosis of solid masses and in difficult-to-access anatomic sites, as well as in mediastinal lymph nodes and pancreatic tumors. The procedure is based on the degree of tissue elasticity measurement, with a good correlation between the elasticity index and histopathological features. We report the case of four patients evaluated by echoendoscopy and qualitative elastography who had differential diagnoses in mediastinal lymph nodes: sarcoidosis, lymphoma, histoplasmosis and esophageal neoplasia.

## INTRODUCTION

Endoscopy ultrasonography (EUS) allows the evaluation of tissues and digestive tract organs, and adjacent structures. Because of the precision of high-resolution images, this type of procedure became a diagnostic marker and widely used for management of treatment of mediastinal and abdominal diseases. However, the solely use of EUS has limitations to determine etiology of an injury, and in some cases, there is need to conduct fine-needle aspiration punctures (FNAP) to confirm malignity.

The EUS-FNAP presents 85% sensibility and 100% specificity for diagnosis of mediastinal lymphadenomegaly.^(^^1^^)^ Despite of this good performance, it is an operator-dependent method. In specific cases, there is need to repeat punctures due to false-negative results, especially for the growing incidence of granulomatous diseases.^(^^2^^)^ In case of multiple suspect lymph nodes is not always is simple to decide to perform puncture.

Elastography, a widely diffused procedure in conventional ultrasonography, was recently incorporated in echoendoscopy. This is an innovative and promising technology that has the goal the increase predictive negative goal of EUS-FNAP.^(^^3^^)^ This is useful to direct puncture in suspect areas and, consequently, improvement of diagnosis performance. This is an easy-to-perform, non-invasive technique with no additional cost and complications.

Main indications for this technique are analysis of solid pancreatic masses, lymph nodes, subepithelial injuries, left liver lobe injuries and left suprarenal. Negative or inconclusive cases of FNAP can be submitted to elastography, when there is a strong suspicion of malignancy.^(^^3^^)^ In addition, EUS-FNAP has high precision for differential diagnosis of solid masses and difficult to reach anatomical sites, such as mediastinal lymph nodes and pancreatic tumors.^(^^4^^)^

The elastography is fundamental for analysis of tissue elasticity degree, which is evaluated by deformation of structures after compression in ultrasonography image in B mode. The qualitative technology is based in detection of these images taken by process with specific software.

Little structural deformations in the image are smaller in hard tissues than in soft tissues. By convention, values of elasticity are represented by color maps (red, yellow, green and blue), in rigid tissues area values are represented by blue, areas with intermediate elasticity by green and yellow, and soft tissues by red.^(^^2^^)^ Consequently, tissues with suspicion of malignancy tends to be represented by blue-like, whereas granulomatous or inflammatory by green-like colors.^(^^5^^)^

Quantitative elastography uses the technology called “shear-wave elasticity imaging” that applies dynamic tension to generate deformation in parallel or perpendicular dimension lines. Speed measuring of shear-wave elasticity results in qualitative and quantitative estimations of tissue elasticity.

There are three types of techniques for shear-wave elasticity imaging: 1- unidimensional transient elastography (1D-TE), 2- elastography of qualitative specific shear wave (pSWE), and 3- elastography of quantitative bidimensional shear wave (2D-SWE).^(^^6^^)^

A good correlation exists between elasticity index and histopathological characteristics, showing sensibility, specificity, positive and negative predictive3 values, and diagnostic accuracy of 100%, 92.3%, 94.6%, 100%, and 96.7%, respectively, in lymphomegalies.^(^^3^^)^

We report the case of four patients who underwent echoendoscopy with pSWE and FNAP with different diagnosis.

## CLINICAL REPORTS

### Report 1: Histoplasmosis

A 42-year-old man with chest pain, dysphagia and coughing. The computed tomography exam revealed augmentation of mediastinal lymph nodes.

High digestive endoscopy showed bulging in left lateral wall of esophagus with elevated fusiform injury and peak erosion. Extension at 25 to 40cm from the incisors.

**Figure 1:**
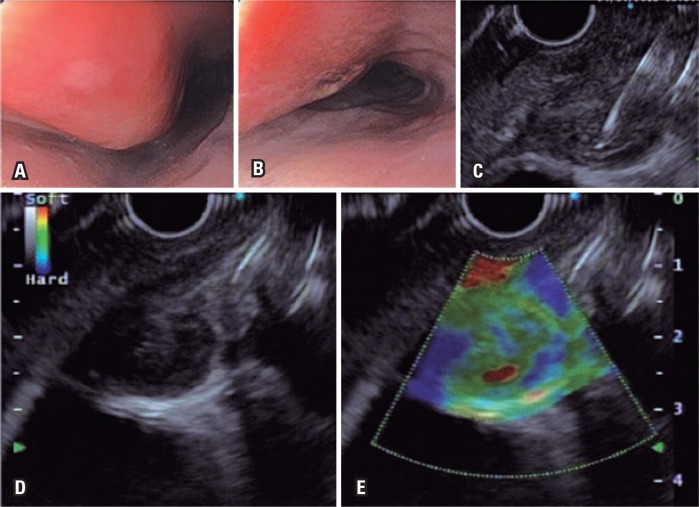
High digestive endoscopy and echoendoscopy. (A and B) Lateral wall bulging of esophagus wall, with elevated fusiform injury and erosion; (C) Echoguided puncture of mediastinal lymph node; (D) Hypoechogenic lymph node, heterogenous, with hyperechogenic center, and hypervascularization showed in Doppler ultrasound (high flow) measuring around 15mm; (E) Qualitative elastography with soft characteristics (green-like)

The EUS presented sectorial probe with interchangeable frequencies of 7.5MHz up to 12.0MHz for the lymphadenomegaly investigation which was observed in a previous imaging exam. Left lateral wall of esophagus was thickened with 11mm and without common echography stratification in layers measuring 15cm longer until the esophagogastric transition. In addition, we observed lymphadenomegaly along paraesophageal chains, aortopulmonary window and subcarinal, in which the majority of them were triangle, hypoechogenic, heterogenous, with hyperechogenic center, and hypervascularization showed in Doppler ultrasound (high flow); the largest measured 15mm. Qualitative elastography with benign characteristic (green-like). Some lymph nodes had contiguity with esophagus wall (Figure 1). Echoguided punctures were conducted.

Anatomopathological study showed epithelioid granuloma, and central necrosis areas. Moderate inflammatory infiltrate in circumjacent mix. Chronic inflammatory process granulomatous with necrosis. Acid-alcohol-resistant negative bacillus (BAAR; Ziehl-Neelsen).

Fungal was study by positive Grocott staining for different sized yeast. Serum dosage of antibodies (complement fixation) for histoplasmosis with titer 1:32 (titer reference: positive if higher than 1:8).

### Report 2: Lymphoma

An 82-year-old woman with coughing, weight loss and asthenia. Her computed tomography revealed increased mediastinal lymph nodes.

The EUS showed sectorial probe with interchangeable frequencies of 7.5MHz up to 12.0MHz for mediastinal assessment.

**Figure 2:**
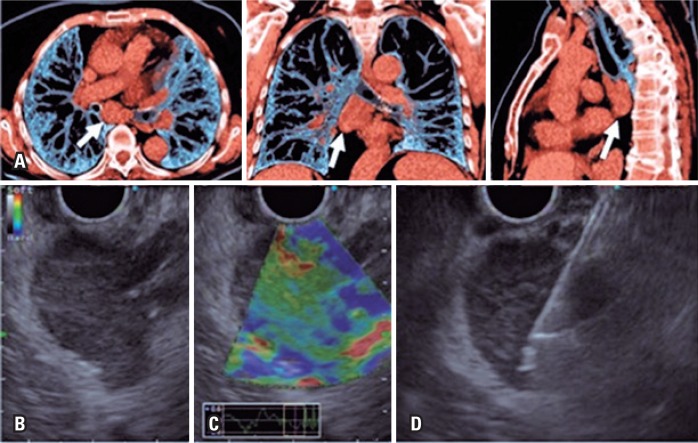
Subcarinal lymph node. (A) Reconstruction of chest tomography in axial, coronal and sagittal cuts, with increased subcarinal lymph node (white arrow); (B) Hypoechogenic lymph node, homogenous, with hyperechogenic, measuring around 30mm; (C) Qualitative elastography with benign characteristic (green axis); (D) Echoguided puncture

Well-defined, rounded, homogenous and hypoechogenic lymph nodes were present in low left paratracheal lymph nodes; the largest measuring 6mm in lower axis and subcarinal region that measured up to 33×30mm. Doppler ultrasound did not reveal larger caliber lymphatic vessels . Qualitative elastography showed soft consistence (Figure 2).

Echoguided puncture were conducted without intercurrences along subcarinal chains.

Cytology indicated atypical lymphoid cells of large nucleus, multinucleate, evident nucleolus, and scarce cytoplasm, in macrophages and other smaller lymphocytes. Granulomas were lacking. Positive neoplastic cells, similar to B-cell Non-Hodgkin lymphoma, positive CD20 with immunoexpression of associated antigens to Epstein-Barr virus.

### Report 3: Esophagus neoplasia

A 50-year-old man who underwent esophagectomy due to cancer. The patient was followed-up because of presence of mediastinal left paratracheal lymph node and increased FDG metabolism (Standard uptake value - SUV= 5.5) revealed in positron-computed tomography emission.

The EUS presented sectorial probe with interchangeable frequencies of 7.5MHz up to 12.0MHz in the remnant esophagus in lymphadenomegaly investigation observed in a previous imaging exam.

**Figure 3:**
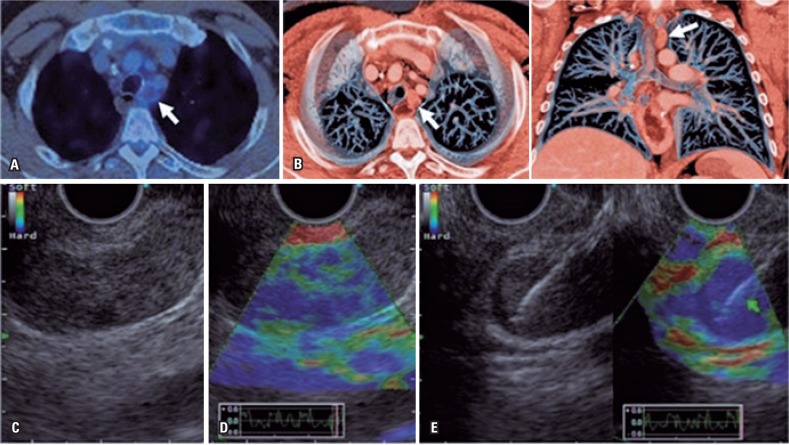
Paratracheal lymph node. (A) positron tomography emission imaging with left paratracheal lymph node (SUV 5.5); (B) Reconstruction in chest tomography in axial and coronal cuts, with increased lymph nodes (white arrow); (C) Endoscopic ultrasound showed hypoechogenic, homogenous lymph node measuring around 25mm; (D) Qualitative elastography with intermediary rigidity characteristics (blue and green); (E) Echoguided puncture

In topography of left superior paratracheal region, we observed longed, rounded, hypoechogenic, homogenous lymph node with well-defined edges, measuring 25mm. The qualitative elastography resources revealed intermediary rigidity (blue and green). We conducted PAAF of 22G (Figure 3).

Anatomopathological study revealed cell block with high cellularity, represented by atypical epithelial cells, and mucosecreting available in tubular and solid arrangements. We observed a single metastatic adenocarcinoma.

### Report 4: Sarcoidosis

A 37-year-old man reporting chest discomfort and dyspnea during efforts. His chest tomography exam revealed discrete diffuse pulmonary infiltrated and increased mediastinal lymph node and hillers.

**Figure 4:**
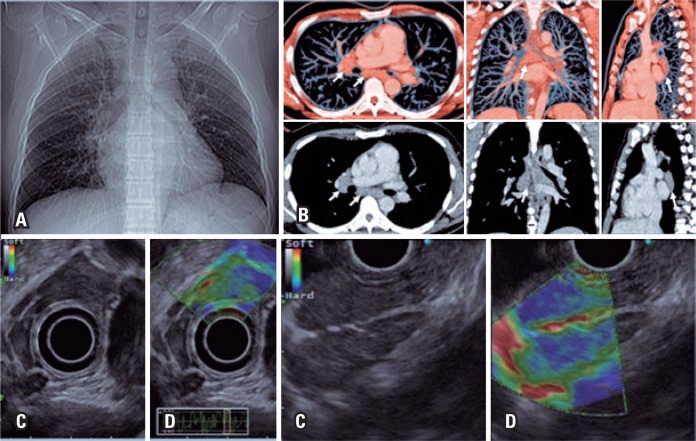
Mediastinal enlargement. (A) Chest radiograph; (B) Reconstruction of chest tomography in axial, coronal and sagittal cuts, with increased lymph nodes (white arrows); (C) Endoscopic ultrasound showed hypoechogenic, homogenous, lymph nodes measuring around 25mm; (D) Qualitative elastography with benign characteristics (green-like)

The EUS presented radial and sectorial probe with interchangeable frequencies of 7.5MHz up to 12.0MHz. We observed a number of hypoechogenic, well-delineated homogenous lymph nodes – some rounded, others long, the greatest of them measured 25mm -, and were located mainly in subcarinal and para-aortic region. The quantitative elastography showed heterogeneous standard, with predominance of blue and green colors. Echoguided punctures were conducted (Figure 4).

Anatomopathological study indicated limph-hystiocitary cells within erythrocytes and with predominance of epithelioid granuloma in which no necrosis was found. Fungal (Grocott) and BAAR (Ziehl-Neelsen) studies were negative. We observed granulomatous chronic inflammation. The diagnosis was sarcoidosis.

## CONCLUSION

Qualitative elastography is method that provides value to echoendoscopy. When associated with fine-needle aspiration puncture, mainly for the analysis of mediastinal lymphadenomegaly, this method can improve the results of histologic analysis. It increases the negative predictive values. The echoendoscopy with qualitative elastography is useful for diagnosis of malignant and benign diseases.
