# Supplementary material for: Echoendoscopy with elastography in mediastinal lymph nodes
Source: Einstein (Sao Paulo). 2019 Nov 27;17(4):eMD5157. doi: 10.31744/einstein_journal/2019MD5157 (PMC6882555; doi:10.31744/einstein_journal/2019MD5157)
Supplement: Translation

# Translation: Ecoendoscopia com elastografia em linfonodos mediastinais

**Authors:** Rogerio Colaiacovo, Altair da Silva Costa, Gustavo Andrade de Paulo, Silvia Mansur Reimão, Marco Antonio Ribeiro Camunha

**Affiliations:** 1 Hospital Israelita Albert Einstein, São Paulo, SP, Brasil; 2 Escola Paulista de Medicina, Universidade Federal de São Paulo, São Paulo, SP, Brasil

**Keywords:** Mediastino, Biópsia por agulha fina, Aspiração por agulha fina guiada por ultrassom endoscópico/métodos, Ultrassonografia de intervenção, Linfonodos, Broncoscopia

## Abstract

A elastografia, procedimento difundido em ultrassonografia convencional, foi recentemente incorporada à ecoendoscopia. Trata-se de tecnologia inovadora e promissora, que visa aumentar o valor preditivo negativo da ultrassonografia endoscópica e das punções aspirativas com agulha fina. É útil para o direcionamento das punções em áreas suspeitas e, consequentemente, melhora o rendimento diagnóstico. Trata-se de técnica não invasiva, de fácil realização, sem custos adicionais ou complicações. As principais indicações são para análise de massas pancreáticas sólidas, linfonodos, lesões subepiteliais, lesões em lobo hepático esquerdo e em suprarrenal esquerda. Casos negativos ou inconclusivos das punções aspirativas com agulha fina podem ser submetidos à elastografia, quando há forte suspeita de malignidade. Tem alta precisão no diagnóstico diferencial de massas sólidas e em locais anatômicos de difícil acesso, como em linfonodos mediastinais e tumores pancreáticos. Baseia-se na quantificação do grau de elasticidade tecidual, com boa correlação entre o índice de elasticidade e as características histopatológicas. Relatamos quatro pacientes avaliados por ecoendoscopia e elastografia qualitativa com diagnósticos diferentes em linfonodos mediastinais: sarcoidose, linfoma, histoplasmose e neoplasia de esôfago.

## INTRODUÇÃO

A ultrassonografia endoscópica (USE) permite a avaliação de tecidos e órgãos do trato digestivo e estruturas adjacentes. Em função da precisão de suas imagens de alta resolução, tornou-se um marco no diagnóstico e no tratamento de doenças mediastinais e abdominais. Isoladamente, possui limitação para determinar a etiologia de uma lesão, sendo necessária, em alguns casos, a realização de punções aspirativas com agulha fina (PAAF) para confirmação de malignidade.

A USE-PAAF apresenta sensibilidade e especificidade de 85% e 100%, respectivamente, em lifonodomegalias mediastinais.^(^^1^^)^ Apesar de seu bom desempenho, é um método operador-dependente. Em casos isolados, é necessária a repetição das punções devido a resultados falso-negativos, principalmente pela alta incidência de doenças granulomatosas em nosso meio.^(^^2^^)^ Na presença de múltiplos linfonodos suspeitos no mediastino posterior, nem sempre é simples a decisão de qual deve ser puncionado.

A elastografia, procedimento amplamente difundido em ultrassonografia convencional, foi, recentemente, incorporada à ecoendoscopia. Trata-se de uma tecnologia inovadora e promissora, que objetiva aumentar o valor preditivo negativo da USE-PAAF.^(^^3^^)^ É útil para o direcionamento das punções em áreas suspeitas e, consequentemente, melhora o rendimento diagnóstico. Trata-se de técnica não invasiva, de fácil realização, sem custos adicionais e sem complicações.

As principais indicações são para análise de massas pancreáticas sólidas, linfonodos, lesões subepiteliais, lesões em lobo hepático esquerdo e em suprarrenal esquerda. Casos negativos ou inconclusivos da PAAF podem ser submetidos à elastografia, quando há forte suspeita de malignidade.^(^^3^^)^ Tem alta precisão no diagnóstico diferencial de massas sólidas e em locais anatômicos de difícil acesso, como em linfonodos mediastinais e tumores pancreáticos.^(^^4^^)^

A elastografia é fundamental na análise do grau de elasticidade tecidual, avaliada pela deformação das estruturas após compressão na imagem ultrassonográfica em modo B. A tecnologia qualitativa é baseada na detecção dessas imagens, captadas por um processador com software específico.

Pequenas deformações estruturais na imagem são menores em tecidos duros do que em tecidos macios. Por convenção, os valores da elasticidade são representados por um mapa de cores (vermelho, amarelo, verde e azul), em que tecidos rígidos são mostrados em tons de azul, os com elasticidade intermediária, em verde e amarelo, e os tecidos macios, em vermelho.^(^^2^^)^ Por conseguinte, tecidos com suspeita de malignidade tendem a ter coloração azulada, enquanto doenças granulomatosas ou inflamatórias expressam tons esverdeados.^(^^5^^)^

A elastografia quantitativa utiliza a tecnologia chamada “onda de cisalhamento da imagem”, que aplica uma tensão dinâmica para gerar deformações nas dimensões paralela ou perpendicular. A mensuração da velocidade da onda de cisalhamento resulta em estimativas qualitativas e quantitativas da elasticidade do tecido. Existem atualmente três tipos de técnicas para imagens de ondas de cisalhamento: elastografia transitória unidimensional (1D-TE), elastografia de onda de cisalhamento pontual qualitativa (pSWE) e elastografia de onda de cisalhamento bidimensional quantitativa (2D-SWE).^(^^6^^)^

É boa a correlação entre o índice de elasticidade e as características histopatológicas, evidenciando sensibilidade, especificidade, valores preditivos positivo e negativo, e acurácia diagnóstica de 100%, 92,3%, 94,6%, 100% e 96,7%, respectivamente, em linfonodomegalias.^(^^3^^)^

Relatamos, a seguir, quatro pacientes submetidos à ecoendoscopia com pSWE e PAAF, com diferentes diagnósticos.

## RELATOS CLÍNICOS

### Relato 1: Histoplasmose

Paciente do sexo masculino, 42 anos, com dor torácica, disfagia e tosse. Tomografia com aumento de linfonodos mediastinais.

A endoscopia digestiva alta mostrou abaulamento na parede lateral esquerda do esofágica, com lesão elevada fusiforme e erosão em seu ápice. Extensão dos 25 até 40cm dos incisivos.

A USE apresentou probe setorial com frequências intercambiáveis de 7,5MHz até 12,0MHz para estudo de linfonodomegalia observada em exame de imagem prévio. A parede lateral esquerda do esôfago estava espessada, com 11mm, e sem a estratificação ecográfica habitual das camadas, com 15cm de extensão, até junto à transição esofagogástrica. Notam-se linfonodomegalias em cadeias paraesofágicas, janela aortopulmonar e subcarinal, a maioria delas triangular, hipoecogênica, heterogênea, com centro hiperecogênico, hipervascularizada ao estudo Doppler (*high flow*), medindo o maior deles 15mm. Elastografia qualitativa com característica benigna (tons esverdeados). Alguns linfonodos apresentavam contiguidade com a parede esofágica (Figura 1). Foram realizadas punções ecoguiadas.

**Figura 1:** Endoscopia digestiva alta e ecoendoscopia. (A e B) Abaulamento na parede lateral esquerda da parede esofágica, com lesão elevada fusiforme e erosão; (C) Punção ecoguiada do linfonodo mediastinal; (D) Linfonodo hipoecogênico, heterogêneo, com centro hiperecogênico, hipervascularizado ao estudo Doppler (*high flow*), tamanho ao redor de 15mm; (E) Elastografia qualitativa com características amolecidas (tons esverdeados)

Anatomopatológico com granulomas epitelioides, com áreas de necrose central. Moderado infiltrado inflamatório misto circunjacente. Processo inflamatório crônico granulomatoso com necrose. Pesquisa de bacilos álcool-ácido resistentes (BAAR; Ziehl-Neelsen) negativa.

Pesquisa de fungos pela coloração de Grocott positiva para leveduras de tamanhos variados. Dosagem sérica de anticorpos (fixação de complementos) para hitoplasmose com titulação 1:32 (referência: positivo se acima de 1:8).

### Relato 2: Linfoma

Paciente do sexo feminino, 82 anos, com tosse, emagrecimento e astenia. Tomografia com aumento de linfonodos mediastinais.

A USE mostrou probe setorial com frequências intercambiáveis de 7,5MHz até 12,0MHz para avaliação mediastinal.

Estavam presentes linfonodos bem definidos, arredondados, homogêneos e hipoecogênicos, nas cadeias linfonodais paratraqueais baixas à esquerda, com o maior medindo 6mm no menor eixo e na região subcarinal, medindo até 33x30mm. A avaliação Doppler não revelou vasos linfonodais de maiores calibres. A elastografia qualitativa mostrou consistência amolecida (Figura 2).

**Figura 2:** Linfonodo subcarinal. (A) Reconstrução da tomografia de tórax em cortes axial, coronal e sagital, com aumento de linfonodo subcarinal (seta branca); (B) Linfonodo hipoecogênico, homogêneo, com centro hiperecogênico, tamanho ao redor de 30mm; (C) Elastografia qualitativa com características benignas (tons esverdeados); (D) Punção ecoguiada

Foram realizadas punções ecoguiadas sem intercorrências, na cadeia subcarinal.

Citologia indicou células linfoides atípicas de núcleos grandes, multinucleados, nucléolos evidentes e citoplasma escasso, em meio a macrófagos e outros linfócitos pequenos. Granulomas estavam ausentes. Positivo para células neoplásicas, compatível com linfoma não Hodgkin de células B, CD20 positivo com imunoexpressão de antígenos associados ao vírus Epstein-Barr.

### Relato 3: Neoplasia de esôfago

Paciente do sexo masculino, 50 anos, pós-operatório de esofagectomia por neoplasia, no acompanhamento por presença de linfonodo mediastinal paratraqueal esquerdo, com aumento do metabolismo de FDG (Standard uptake value - SUV= 5,5) na tomografia por emissão de pósitron - tomografia computadorizada.

A USE indicou probe setorial com frequências intercambiáveis de 7,5MHz até 12,0MHz no remanescente esofágico, para estudo de linfonodomegalia observada em exame de imagem.

Na topografia da região paratraqueal superior esquerda, notou-se linfonodo alongado, ovalado, hipoecogênico, homogêneo, de limites bem definidos, que media 25mm. O recurso da elastografia qualitativa revelou rigidez intermediária (azulado e esverdeado). Foi realizadas PAAF de 22G (Figura 3).

**Figura 3:** Linfonodo paratraqueal. (A) Imagem de tomografia por emissão de pósitron com linfonodo paratraqueal esquerdo (SUV 5,5); (B) Reconstrução da tomografia de tórax em cortes axial e coronal, com aumento de linfonodo (seta branca); (C) Ultrassonografia endoscópica mostra linfonodo hipoecogênico, homogêneo, com tamanho ao redor de 25mm; (D) Elastografia qualitativa com características de rigidez intermediária (azulado e esverdeado); (E) Punção ecoguiada

Anatomopatológico apresentou emblocado com celularidade alta, representado por células epiteliais atípicas, por vezes mucossecretoras, dispostas em arranjo tubular e sólido. Observou-se um adenocarcinoma metastático.

### Relato 4: Sarcoidose

Paciente do sexo masculino, 37 anos, com desconforto torácico e dispneia aos esforços. Tomografia de tórax com infiltrado pulmonar discreto difuso e aumento de linfonodos mediastinais e hilares.

A USE expôs probes radial e setorial com frequências intercambiáveis de 7,5MHz até 12,0MHz. Notavam-se vários linfonodos hipoecogênicos, homogêneos, de limites bem definidos – alguns arredondados, outros alongados; o maior deles media cerca de 25mm –, localizados, principalmente, em região subcarinal e região para-aórtica. A elastografia qualitativa evidenciou padrão heterogêneo, com predomínio das cores azul e verde. Foram realizadas punções ecoguiadas (Figura 4).

**Figura 4:** Alargamento mediastinal. (A) Radiografia de tórax; (B) Reconstrução da tomografia de tórax em cortes axial, coronal e sagital, com aumento de linfonodos (setas brancas); (C) Ultrassonografia endoscópica mostra linfonodos hipoecogênicos, homogêneos, tamanho maior ao redor de 25mm; (D) Elastografia qualitativa com características benignas (tons esverdeados)

Anatomopatológico indicou células linfo-histiocitárias em meio a hemácias e com predomínio de granulomas epitelioides, nas quais não se identifica necrose. As pesquisas de fungos (Grocott) e de BAAR (Ziehl-Neelsen) foram negativas. Observou-se inflamação crônica granulomatosa. O diagnóstico foi de sarcoidose.

## CONCLUSÃO

A elastografia qualitativa é um método que agrega valores à ecoendoscopia. Quando associada às punções aspirativas com agulhas finas, principalmente na análise de linfonodomegalias mediastinais, pode incrementar os resultados da análise histológica. Há um aumento do valor preditivo negativo. O método é útil para diagnóstico nas doenças malignas e benignas.
